# Supplementary material for: Hybrid Models and Biological Model Reduction with PyDSTool
Source: PLoS Comput Biol. 2012 Aug 9;8(8):e1002628. doi: 10.1371/journal.pcbi.1002628 (PMC3415397; doi:10.1371/journal.pcbi.1002628)
Supplement: Text S4 — Complete source code for the PyDSTool package (version 0.88.120504). Includes API documentation and help files linking to web pages. This file is identical to the current public release on Sourceforge.net. (ZIP) [file pcbi.1002628.s004.zip › PyDSTool/html/toc-PyDSTool.utils-module.html]

xml version="1.0" encoding="ascii"?


utils


# Module utils

---

## Functions

cartesianProduct  
 compareList  
 find  
 findClosestArray  
 findClosestPointIndex  
 info  
 intersect  
 loadObjects  
 makeDataDict  
 makeImplicitFunc  
 makeMfileFunction  
 make\_Jac\_wrap  
 make\_RHS\_wrap  
 orderEventData  
 remain  
 saveObjects  
 union  

## Variables

API

API\_class  

Continuous

Discrete

DomainType  

Inf

LargestInt32

Macheps  

NaN

\_1DimplicitSolveMethods  

\_all\_complex

\_all\_float

\_all\_int

\_all\_numpy\_complex

\_all\_numpy\_float

\_all\_numpy\_int

\_classes

\_complex\_types

\_float\_types

\_functions

\_implicitSolveMethods  

\_int\_types

\_mappings

\_num\_equivtype

\_num\_maxmin

\_num\_name2equivtypes

\_num\_name2type

\_num\_type2name

\_num\_types

\_pytypefromtype

\_real\_types

\_seq\_types

all  
 any  
 argmax  
 atleast\_1d  
 clip  
 complex  
 complex128  
 complex192  
 complex64  
 complex\_  
 complexfloating  
 deepcopy  
 double  
 exp  
 finfo  
 fit\_function  
 float  
 float32  
 float96  
 float\_  
 floating  

greater

int  
 int0  
 int16  
 int64  
 int8  
 int\_  
 integer  

isfinite

less

less\_equal  
 log  
 logical\_or  
 mat  
 npy  

null\_predicate

null\_predicate\_class  
 ones  
 predicate\_op  
 putmask  

rerr

rout

shape  
 sign  
 spy  
 sqrt  

targetLangs

types  

---

[hide private]
